# A new sensitive PCR assay for one-step detection of 12 IDH1/2 mutations in glioma

**DOI:** 10.1186/2051-5960-2-58

**Published:** 2014-06-02

**Authors:** Aurélie Catteau, Hélène Girardi, Florence Monville, Cécile Poggionovo, Sabrina Carpentier, Véronique Frayssinet, Jesse Voss, Robert Jenkins, Blandine Boisselier, Karima Mokhtari, Marc Sanson, Hélène Peyro-Saint-Paul, Caterina Giannini

**Affiliations:** QIAGEN Marseille, Av. de Luminy, Marseille, France; AP-HP, Pitié-Salpêtrière Hospital, Paris, France; Mayo Clinic, Rochester, Rochester, MA USA

**Keywords:** Glioma, IDH1/2, Quantitative real-time PCR

## Abstract

**Introduction:**

Mutations in isocitrate dehydrogenase genes *IDH1* or *IDH2* are frequent in glioma, and IDH mutation status is a strong diagnostic and prognostic marker. Current IDH mutation screening is performed with an immunohistochemistry (IHC) assay specific for IDH1 R132H, the most common mutation. Sequencing is recommended as a second-step test for IHC-negative or -equivocal cases. We developed and validated a new real-time quantitative polymerase chain reaction (PCR) assay for single-step detection of IDH1 R132H and 11 rare IDH1/2 mutations in formalin-fixed paraffin-embedded (FFPE) glioma samples. Performance of the IDH1/2 PCR assay was compared to IHC and Sanger sequencing.

**Results:**

The IDH1/2 PCR assay combines PCR clamping for detection of 7 IDH1 and 5 IDH2 mutations, and Amplification Refractory Mutation System technology for specific identification of the 3 most common mutations (IDH1 R132H, IDH1 R132C, IDH2 R172K). Analytical sensitivity of the PCR assay for mutation detection was <5% for 11/12 mutations (mean: 3.3%), and sensitivity for mutation identification was very high (0.8% for IDH1 R132H; 1.2% for IDH1 R132C; 0.6% for IDH2 R172K). Assay performance was further validated on 171 clinical glioma FFPE samples; of these, 147 samples met the selection criteria and 146 DNA samples were successfully extracted. IDH1/2 status was successfully obtained in 91% of cases. All but one positive IDH1 R132H-IHC cases were concordantly detected by PCR and 3 were not detected by sequencing. Among the IHC-negative cases (n = 72), PCR detected 12 additional rare mutations (10 IDH1, 2 IDH2). All mutations detected by sequencing (n = 67) were concordantly detected by PCR and 5/66 sequencing-negative cases were PCR-positive (overall concordance: 96%). Analysis of synthetic samples representative of the 11 rare IDH1/2 mutations detected by the assay produced 100% correct results.

**Conclusions:**

The new IDH1/2 PCR assay has a high technical success rate and is more sensitive than Sanger sequencing. Positive concordance was 98% with IHC for IDH1 R132H detection and 100% with sequencing. The PCR assay can reliably be performed on FFPE samples and has a faster turnaround time than current IDH mutation detection algorithms. The assay should facilitate implementation of a comprehensive IDH1/2 testing protocol in routine clinical practice.

**Electronic supplementary material:**

The online version of this article (doi:10.1186/2051-5960-2-58) contains supplementary material, which is available to authorized users.

## Introduction

Histopathological evaluation remains the gold standard for glioma classification [1] but the incorporation of emergent molecular biomarkers has been shown to improve diagnosis and prognosis of this heterogeneous disease. In addition to the established 1p/19q co-deletion and *MGMT* methylation, new biomarkers including *IDH1/2, EGFR* or *BRAF* mutations and *FGFR* gene fusions, are increasingly documented to play a role as diagnostic, prognostic or predictive markers, and should progressively be introduced in the diagnostic and treatment decision algorithm for glioma [2, 3].

*IDH1/2* mutations are highly frequent (up to 80%) in diffuse glioma [4–6]. Their identification in surgical neuropathology samples increases diagnostic accuracy of World Health Organization (WHO) grade II or III astrocytoma, oligodendroglioma, oligoastrocytoma, and WHO grade IV secondary glioblastoma (GBM) [5, 7]. In addition, *IDH* mutations have been repeatedly shown to be associated with significantly better patient survival, thereby providing valuable prognostic information [8–12]. Based on these findings, *IDH1* mutation status is becoming part of the standard diagnostic assessment of these tumors and will likely be included in the next WHO classification of diffuse gliomas [2].

In addition, *IDH* mutation status may predict benefit from alkylating agent when combined with *MGMT* promoter methylation assessment [13]. Very recently, updated data from the RTOG 9402 trial showed that the *IDH* mutation predicts the benefit of adjuvant chemotherapy in grade III glioma, even in absence of 1p19q co-deletion [14]. Recent data suggested that *IDH1* mutation may also serve as a predictive marker to guide aggressive surgical resection of malignant astrocytomas [15].

Given the high IDH clinical relevance, stratification according to *IDH* mutation should be taken into account for more effective future clinical trials [2, 16]. In addition, the discovery of *IDH* mutations has led to the development of novel therapies targeted against IDH alterations, using selective IDH inhibitors or by reversion of mutated-IDH induced hypermethylation with the use of DNA methyltransferase inhibitors [17–19].

*IDH1* R132H mutation represents the most common *IDH* mutation (approximately 90%). Less common are the *IDH* mutations within the same *IDH1* codon 132 (around 7%), and in the homologous *IDH2* codon 172 (approximately 3%) [20].

An algorithm for *IDH* mutation screening has recently been proposed [21]. It is a two-step process implying initial search for the most common IDH1 R132H mutation using immunohistochemistry (IHC)-based assay, followed by DNA-based analysis on IHC-negative or -equivocal cases. The IDH1 R132H mutation-specific antibody shows high sensitivity and specificity [7, 22], but the IHC technique can be problematic in some cases, as a result of background staining or regional heterogeneity of IDH1 R132H protein expression [23]. Regarding DNA-based analyses, Sanger sequencing still represents the gold standard for the identification of somatic mutations. However, sequencing sensitivity is low (around 15-20% mutation load) and this may lead to false negative results when analyzing tumor specimens with insufficient neoplastic cells in a background of normal cells. Moreover, this technology is also not readily available in all neuropathological centers, and its use generally leads to additional delay in providing a comprehensive *IDH* mutational status assessment. Here we describe a new *IDH1*/2 polymerase chain reaction (PCR) assay which was designed to detect the most common *IDH1* R132H mutation and 11 rare *IDH* mutations in one single step using formalin-fixed paraffin-embedded (FFPE) samples. Analytical studies were conducted to verify its performance. This assay was further validated on a large series of FFPE glioma samples to determine the reliability of the method in a routine clinical setting, by comparing *IDH1*/*2* mutation analysis with IHC and Sanger sequencing.

## Materials and methods

### *IDH1/2* one-step quantitative PCR assay

PCR-clamping was used for the qualitative detection of 6 mutations within *IDH1* codon 132 (the 2 major R132H and R132C mutations, and 4 “IDH1-other”: R132G, R132S, R132L, R132V), one within *IDH1* codon 100 (R100Q) and 5 within *IDH2* codon 172 (the major R172K and 4 “IDH2-other”: R172M, R172W, R172S, R172G). Amplification Refractory Mutation System (ARMS) PCR technology was combined to selectively identify the most frequent IDH1 R132H/R132C and IDH2 R172K mutations (Table 1).

**Table 1:** IDH1/2 mutations detected and identified* with the IDH1/2 PCR assay

Gene	Mutation	Base change	Cosmic ID**
*IDH1*	Arg132His (R132H)*	395G > A	COSM28746
	Arg132Cys (R132C)*	394C > T	COSM28747
	Arg132Ser (R132S)	394C > A	COSM28748
	Arg132Gly (R132G)	394 C > G	COSM28749
	Arg132Leu (R132L)	395G > T	COSM28750
	Arg132Val (R132V)	394_395 CG > GT	COSM28751
	Arg100Gln (R100Q)	299 G > A	COSM88208
*IDH2*	Arg172Lys (R172K)*	515G > A	COSM33733
	Arg172Met (R172M)	515G > T	COSM33732
	Arg172Trp (R172W)	514A > T	COSM34039
	Arg172Ser (R172S)	516G > T	COSM34090
	Arg172Gly (R172G)	514A > G	COSM33731

*Mutations identified by ARMS.

**http://www.sanger.ac.uk/genetics/CGP/cosmic.

**Figure 1:**
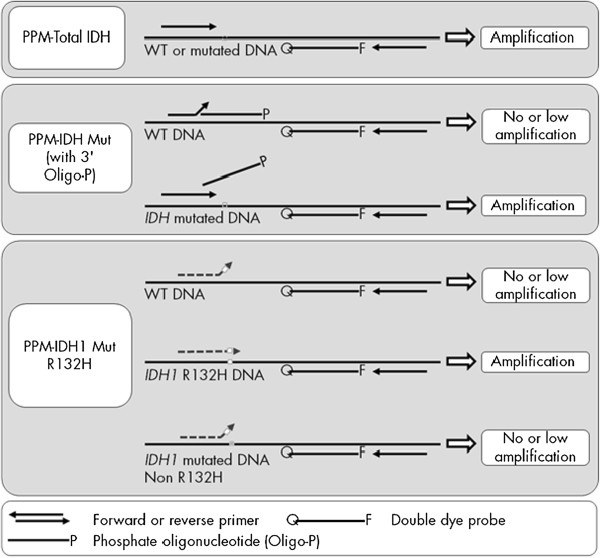
**Principle of the IDH1/2 PCR assay.** Total reaction mixes (top) The total Primers and Probe Mixes (PPM-Total) used primers and probes to amplify both mutated and wild-type (WT) target sequences. Mutation detection reaction mixes (middle) The mutation detection primers and probe mixes combined primers and probes, to amplify both mutated and WT target sequences, plus an oligonucleotide, 3'-blocked with the addition of a phosphate group (3’-Oligo-P) to prevent elongation (PCR clamping), which was specific to the WT target sequence. When the PCR template contained the WT sequence, the 3'-Oligo-P bound preferentially over the PCR primer binding due to higher affinity. There was no or low extension by the DNA polymerase and no or low amplification was observed. When a mutated sequence was present, PCR primer bound preferentially over the 3'-Oligo-P and amplification proceeded. Mutation identification reaction mixes (bottom) Allele-specific amplification was achieved by ARMS® (Amplification Refractory Mutation System), which exploits the ability of the DNA polymerase to distinguish between a match and a mismatch at the 3' end of a PCR primer. When the PCR primer fully matched, the amplification proceeded with full efficiency. When the 3' base was a mismatch, only low-level background amplification occurred. The same principle shown on the figure to detect IDH1 R132H applied for IDH1 R132C and IDH2 R172K.

The *therascreen* IDH1/2 RGQ PCR kit was used following manufacturer’s instructions (Qiagen). The test consisted in 9 separate amplification reactions: (i) 3 total amplification reactions (“Total IDH1 R132”, “Total IDH2 R172” and “Total IDH1 R100”), (ii) 3 mutation amplification reactions of *IDH1* codon 132 (“IDH1 R132 Mut”), *IDH1* codon 100 (“IDH1 R100 Mut”) and *IDH2* codon 172 (“IDH2 R172 Mut”), and (iii) 3 mutation-specific amplification reactions of IDH1 R132H (“IDH1 Mut R132H”), IDH1 R132C (“IDH1 Mut R132C”) and IDH2 R172K (“IDH1 Mut R172K”). Total reaction mixes for (i) contained primers and probes amplifying both wild-type (WT) and *IDH*-mutated target sequences. Mutation detection reaction mixes for (ii) combined specific primers and probes to amplify both mutated and WT target sequences, plus a WT-specific 3’P-blocked oligonucleotide preventing elongation (PCR clamping). Mutation identification for (iii) was achieved by allele-specific amplification using ARMS (Figure 1).

PCRs were performed with 25 ng of sample or control DNA using the Rotor-Gene Q 5-plex HRM instrument (Qiagen). Run controls (positive, negative and no template control) were assessed to ensure that acceptable Ct values were met and that the reactions were performed correctly. Each sample was processed once per PCR run.

Sample ΔCt values were calculated as the difference between the mutation assay Ct and respective total assay Ct from the same sample. Samples were classified as mutation-positive if the ΔCt value was less than or equal to the ΔCt cut-off value of the respective mutation assay.

### Determination of the cut-off values of the assay

Limit of Detection (LOD), defined as the lowest amount of mutant DNA in a WT DNA background, was based on the “precision profile approach” following the CLSI EP17-A2 guideline [24]. A sample set was developed by mixing synthetic IDH mutant DNA with WT genomic DNA to correspond to 5 *IDH1/2* mutation positive percentages (2, 5, 10, 15 and 20%). In total, 30 to 110 measurements were made per mutation type and mutation percentage. Each sample was run in duplicate, over 5 days on 3 different instruments and with 3 different lots of IDH1/2 *therascreen* kits, leading to a total of 2250 ΔCt measurements.

For each mutation LOD, the associated mutation percentage was estimated by fitting a non-linear model between ∆Ct values and mutant percentage.

### *IDH1/2* quantitative PCR assay repeatability and reproducibility

The precision of the *therascreen* IDH1/2 kit was determined using a protocol based on the CLSI EP05-A2 [25]. The following deviations were estimated: within-run repeatability (Sr), between-runs reproducibility (Srun), within and between-days and precision of the assay (Stot).

Synthetic mutant samples were used for this evaluation: WT genomic DNA from clinical glioma FFPE samples was spiked in 3 mastered proportions (5, 10 and 30% mutant copies) with either R100Q, R132C, R132H or R172K plasmid mutant DNA. Corresponding WT DNA (0%) was also tested. Each sample was tested 40 times in duplicate i.e. 80 ΔCt measurements. Runs were performed by 3 operators over 10 days, on 2 RGQ instruments, with 3 different lots of IDH1/2 *therascreen* kits leading to a total of 2240 ΔCt measurements. A variance Component Analysis (fully nested Model II ANOVA) was performed on ΔCt values to estimate the different sources of variability for each mutation test.

### Glioma clinical samples

A total of 171 FFPE clinical tumor samples were collected. In total, 121 samples were retrospectively collected from the Pitié-Salpêtrière Hospital (n = 19) and the Mayo Clinic (n = 102) tumor banks and 50 additional FFPE samples were collected from commercial tumor banks. All FFPE sections were reviewed by local neuropathologists for diagnosis of glioma and assessment of neoplastic cellularity. Samples selection criteria were based on age of the samples (<10 years old), tissue area (≥50 mm^2^) and neoplastic cellularity (sections with ≥40% tumor cells).

### Synthetic mutant-*IDH* DNA samples

Synthetic samples representative of each of the 6 minor *IDH1* and 5 *IDH2* mutations (Table 1) were prepared at 2 dilutions (30 and 40%) by mixing mutant-IDH synthetic DNA into WT *IDH* genomic DNA. The samples were processed similarly to clinical samples.

### Immunochemistry

The presence and extent of tumor was assessed on H&E slides. Immunohistochemical staining for mutant IDH1 was performed using the anti-human IDH1 R132H mouse monoclonal antibody DIA clone H09 (Dianova, Germany) at a dilution of 1:100. Pretreatment of slides with Cell Conditioner 1 (EDTA) MILD using the Ventana BenchMark XT stainer system was followed by incubation with the primary antibody for 32 minutes at 37°C, which was then detected using Ventana UltraView detection with Ventana DAB. Hematoxylin counterstain was performed with the Leica ST 5020 apparatus. Positive and negative controls were performed using specimens previously validated.

### DNA extraction from FFPE samples

DNA was extracted from 10 μm FFPE sections using the QIAamp DNA FFPE Tissue kit (Qiagen) following instructions for use of the *therascreen* IDH1/2 RGQ PCR kit (Qiagen).

### IDH1 and IDH2 sequencing

Bidirectional Sanger sequencing was performed centrally (Qiagen, Hilden). Fragments of 129 base pairs (bp) for *IDH1* codon 132 and 150 bp length for *IDH2* codon 172 were amplified using primers previously described [21]. A fragment containing *IDH1* codon 100 was amplified using the following primers: 5’-AAGGATGCTGCAGAAGCTATAA-3’ (forward) and 5’-CCATAAGCATGACGACCTATGA-3’ (reverse). Briefly, PCR was performed in a total volume of 60 μL using 15 ng of DNA and Taq Polymerase Platinium in standard PCR buffer conditions. Initial denaturation at 94°C for 2 minutes was followed by 35 cycles of 94°C for 30 secondes, 55°C for 30 secondes and 68°C for 30 secondes. PCR products were purified using the QIAquick 96 PCR Purification kit (Qiagen, Hilden). The purified PCR products (1.5 μL) were sequenced using Applied Biosystems Big Dye 3.1 dideoxy chain termination sequencing chemistry in conjunction with fluorescent-based capillary sequencers (Model 3730xl Applied Biosystems Division of Perkin Elmer, Foster City, California, USA) for electrophoresis, data collection and base calling.

Pyrosequencing for detection of *IDH1* codon 132 and *IDH2* codon 172 mutations was performed as previously described [26]. Locked Nucleic Acid (LNA) Sanger sequencing was performed in a separate reaction by amplifying the mutation hotspot regions using the pyrosequencing PCR primers and LNA (+T + A + G + G + T + C + G + T + C + A+/3InvdT/) at the final concentration of 70 nM. The PCR product was sequenced using BigDye Terminator v1.1 Sequencing kit (Applied Biosystems; Foster City, CA, USA).

### Statistical analyses

The main objective of the clinical validation study was to calculate the Positive Percent Agreement (PPA) first, between the IDH1/*2* PCR test and IDH1 R132H IHC, and secondly, between the IDH1/2 PCR test and Sanger sequencing. For both comparisons, the aim was to obtain a PPA of at least 90% with a target of 95%, with a lower boundary (Lbound) of 95% confidence interval (CI) ≥90%.

The PPA between the PCR test and the reference methods (IHC and Sanger sequencing) was calculated as the percentage of true positive (TP) i.e. the percentage of cases detected as positive by both the IDH1/2 PCR test and the reference method, according to the following formula: PPA = (100×TP)/(TP + FN), where FN were false-negative cases i.e. negative by PCR when reference method was positive.

Secondary objectives were to calculate the Negative Percent Agreement (NPA) defined as NPA = (100×TN)/(TN + FP) and overall agreement (OA) defined as OA = (TP + TN)/(TP + TN + FP + FN), where TN and FP were true negative and false positive cases, respectively.

## Results

### Determination of sensitivity

**Figure 2:**
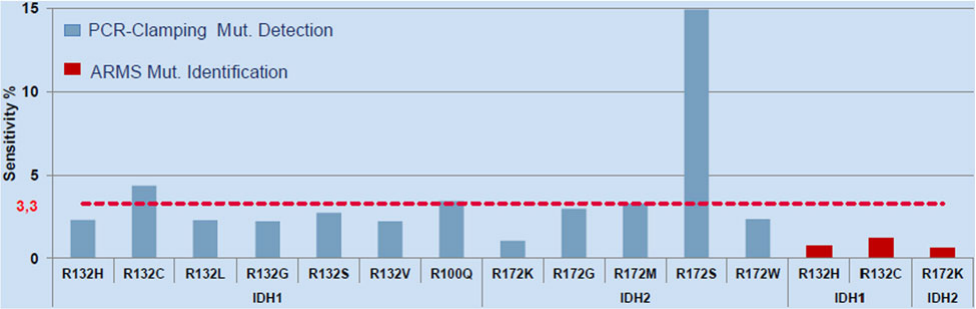
**Sensitivity of the IDH1/2 PCR assay for the 12 detected and 3 identified IDH1/2 mutations.** The sensitivity defined as the lowest amount of mutant DNA in a background of wild-type DNA was indicated for each of the 12 IDH1/2 mutations detected with the PCR IDH1/2 kit. The cut-off values were derived from a total of 2250 ΔCt measurements. Sensitivity varied across mutations from 0.6% to 15% with a mean of 3.3% (red dotted line). Limit of Detection was <5% for 11/12 mutations and ≤3% for 9 of them.

The IDH1/2 assay was based on a combination of PCR-clamping and ARMS technology and can detect 12 *IDH1/2* mutations (Table 1). Assay sensitivity was therefore estimated for each mutation, by LOD using mixed mutant *IDH* DNA with WT genomic DNA at 5 mutation percentages i.e. 2, 5, 10, 15 and 20%. Sensitivity varied according to mutations, ranging from 0.6% to 15%, (Figure 2 and Additional file 1: Table S1) with a mean of 3.3%. Sensitivity results are shown as supplementary data (Additional file 2: Figure S1). Reproducible and reliable mutation detection was achieved at a concentration of mutant DNA below 5% for 11/12 mutations and ≤3% for 9 mutations. The identification of the 3 major *IDH1/2* mutations showed very high sensitivity levels, respectively 0.8% for IDH1 R132H, 1.2% for IDH1 R132C and 0.6% for IDH2 R172K (Figure 2 and Additional file 1: Table S1).

### Repeatability and reproducibility

*IDH* WT and mutant samples were tested at 3 different mutant allele burden (5, 10 and 30%) and precision performance of the IDH1/2 PCR assay was established on a total of 2240 measurements (see Materials and Methods). High precision was demonstrated for the 3 mutant sample levels and WT, with at least 99% correct mutation calls for all mutation assays across multiple lots, platforms and operators for both within- and between-run experiments. Estimates of variance for each tested mutant and WT samples are listed in supplementary Additional file 1: Table S2.

### IDH1/2 PCR assay technical success rate

**Figure 3:**
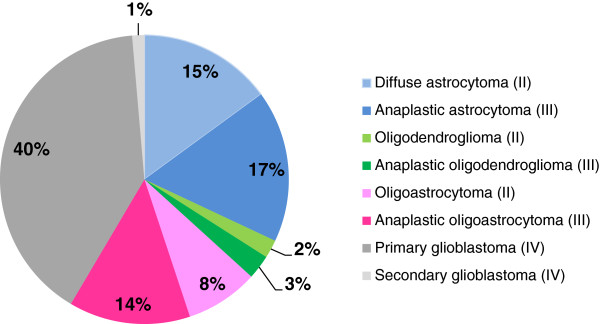
**Distribution of the clinical glioma samples by tumor type (World Health Organization grade) (n = 147).** The majority of the collected formalin-fixed paraffin-embedded clinical samples meeting inclusion criteria for the IDH1/2 PCR test originated from patients with diagnosed primary glioblastoma, the most frequently diagnosed glioma subtype in clinical routine. The remaining samples originated from patients diagnosed with other histological forms, mainly astrocytomas. Samples were available for each diffuse glioma subtype.

**Figure 4:**
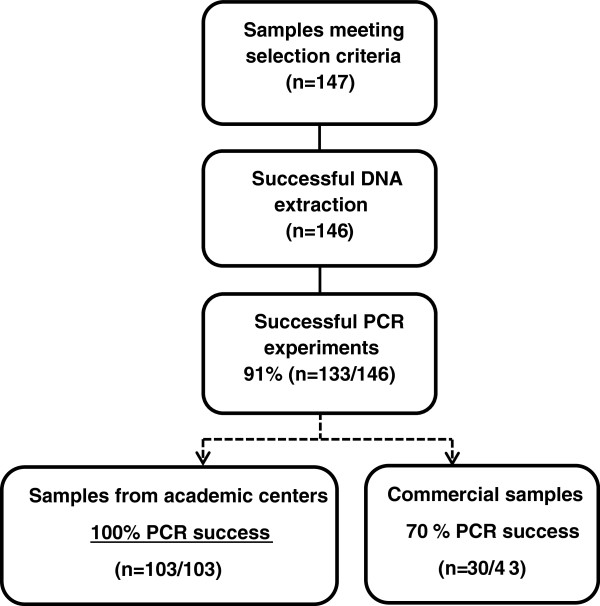
**IDH1/2 PCR assay experimental flowchart: technical success rate.** Out of the 147 formalin-fixed paraffin-embedded glioma clinical samples meeting the selection criteria, one DNA extraction failed. From the remaining samples, IDH1/2 PCR assay results were successfully obtained for most of the samples (91%). Successful tests were achieved on all samples (n = 103) originating from academic centers.

Performance of the assay was established on 171 retrospectively tested FFPE clinical glioma samples collected in 3 different sources: the Pitié-Salpêtrière Hospital, the Mayo Clinic and commercially available. Twenty-four samples did not meet the selection criteria (see Materials and Methods): for 2 cases, diagnosis of glioma was not confirmed; 17 samples were more than 10 years old and 5 samples contained less than 40% tumor cells. Histological distribution of the tumor samples reflected distribution observed in clinical routine with a predominance of glioblastomas (n = 61, 41%). The other samples were astro cytomas (n = 22, 15%), anaplastic astrocytomas (n = 25, 17%), oligodendrogliomas (n = 3, 2%), anaplastic oligodendrogliomas (n = 4, 3%), oligoastrocytomas (n = 12, 8%), and anaplastic oligoastrocytomas (n = 20, 14%) (Figure 3).Out of the 147 FFPE samples which met the selection criteria, DNA extraction failed for one sample, while an IDH1/2 result was successfully obtained by the PCR test in 91% of cases (133/146). Different technical success rates were observed according to the origin of the samples with 100% technical success rate on samples from academic centers (103/103) and 70% on commercial samples (30/43) (Figure 4).

### Clinical validation: comparison of IDH1/2 assay with IDH1 R132 immunochemistry and Sanger sequencing

To demonstrate the accuracy of the IDH1/2 PCR assay on clinical routine glioma samples, we examined the concordance between the IDH1/2 PCR results and results obtained with the current IDH1 R132H immunostaining and Sanger sequencing technologies. Out of the 133 samples successfully assessed by PCR, 61 (46%) were WT and 72 (54%) contained an *IDH1/2* mutation. Out of the 72 mutated specimens, 60 (83%) carried the most common IDH1 R132H mutation, while the 12 remaining cases carried rare IDH mutations: IDH1 R132C in 3 cases, IDH1 R132-other in 7 cases, and IDH2 R172K in 1 case and IDH2 R172-other in 1 case. Using the IHC-based assay, 61 cases out of the 133 were scored as IDH1 R132H-positive. All but one IHC-positive cases were concordantly detected as IDH1 R132H by the PCR test and none of the remaining 72 IHC-negative cases were detected as IDH1 R132H-positive by PCR (Table 2), which translated into an OA of 99% [CI 95%, 95.9-99.9] between PCR and IHC (98.4% positive agreement [CI 95%, 91.3-99.7], 100% negative agreement [CI 95%, 94.9-100]).

**Table 2:** **Agreement between IHC and IDH1/2 PCR assay for the detection of IDH1/2 R132H mutation**

		IHC		
		R132H positive	R132H negative	Total
**IDH1/2 PCR**	**R132H**	60	0	60
	**Non-R132H**	1	72	73
	**Total**	61	72	133

IHC: Immunohistochemistry; PCR: Polymerase chain reaction.

When evaluating the 133 specimens by Sanger sequencing, the same mutations were detected by both Sanger sequencing and PCR in 67 cases (58 IDH1 R132H, 2 IDH1 R132C, 6 IDH1 R132-other and 1 IDH2 R172K), while the PCR assay detected 5 additional mutated cases (2 IDH1 R132H, 1 IDH1 R132C, 1 IDH1 R132-other and 1 IDH2 R172-other) (Tables 3 and 4), resulting in an OA of 96% [CI 95%, 91.5-98.4] between PCR and Sanger sequencing with a 100% positive agreement [CI 95%, 94.6-100] and 92% negative agreement [CI 95%, 83.5-96.7]. Predefined concordance targets for both PCR-IHC and PCR-Sanger comparisons were therefore met. Complete analysis of agreement between the IDH1/2 PCR assay and IHC or Sanger sequencing is shown in supplementary Additional file 1: Table S3.

**Table 3:** **Agreement between Sanger sequencing and IDH1/2 PCR assay for the detection of IDH1/2 R132H mutation**

		Sanger sequencing		
		IDH1/2 mutated	IDH1/2 non-mutated	Total
**IDH1/2 PCR**	**IDH1/2 mutated**	67	5	72
	**IDH1/2 non-mutated**	0	61	61
	**Total**	67	66	133

PCR: Polymerase chain reaction.

**Table 4:** **Number and types of IDH 1/2 mutations detected by IHC, Sanger sequencing and PCR (n = 133)**

	IDH1/2 WT	IDH1 mutated				IDH2 mutated		% Mutated cases
		R132H	R132C	R132 **Other**	R100	R172K	R172 **Other**	
**IHC**	72	61	NA	NA	NA	NA	NA	46
**Sanger sequencing**	66	58	2	6*	0	1	0	50
**IDH1/2 PCR**	61	60	3	7	0	1	1	54

IHC: Immunohistochemistry; PCR: Polymerase chain reaction; WT: Wild-type; NA: not applicable.

*3 R132S; 2 R132G; 1 R132L.

**Figure 5:**
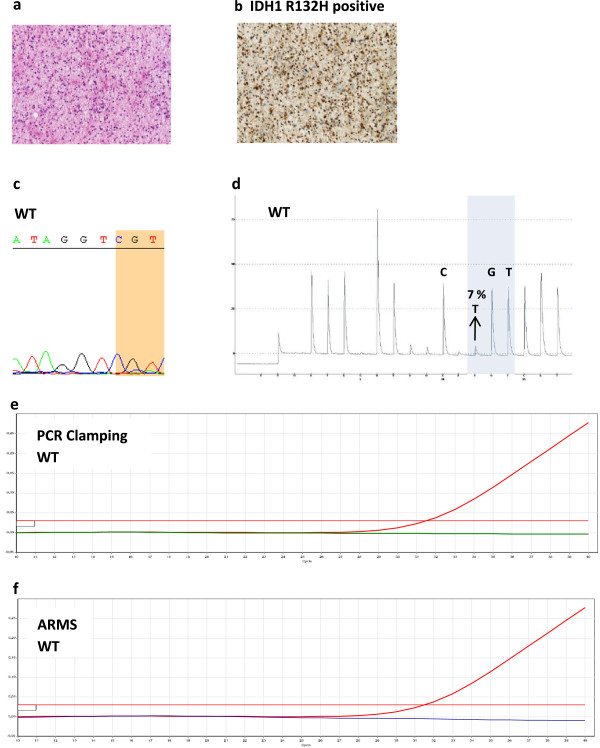
**Discordant case # 1.** Case #1 stained IDH1 R132H positive by immunochemistry but was found WT for the *IDH1* codon 132 (CGT) by Sanger sequencing, by pyrosequencing and with the IDH1/2 PCR assay **a** H&E slide. **b** IHC using the anti-human IDH1 R132H mouse monoclonal antibody DIA clone H09. **c** Sanger sequencing. **d** pyrogram indicating an allele frequency of 7%, thus below the cutoff for mutation ≥ 15%. **e, f** IDH1/2 PCR assay combining PCR-clamping (**e**) and ARMS (**f**): the red horizontal lines correspond to the threshold used to determine Ct. Total copies of *IDH1* (WT ± mutated) are amplified with the total amplification reaction (red curves) but without further amplification by PCR-Clamping (blue curve) or by ARMS with specific R132H primers (green curve), indicating the absence of any mutation within *IDH1* codon 132.

Three IHC-positive cases were not reported positive by Sanger sequencing, including the PCR-negative case. Out of the 72 IHC-negative cases, Sanger sequencing identified 9 rare mutations (12%; 8 IDH1, 1 IDH2) while PCR identified 12 rare mutations (17%; 10 IDH1, 2 IDH2) (Table 4). The 6 cases discordant across the techniques for their identification of IDH mutation status were further investigated (Table 5). Case #1 (Figure 5), a sample of commercial origin, was IDH1 R132H IHC-positive but mutation negative by PCR and Sanger sequencing. We used 2 other techniques more sensitive than Sanger Sequencing: pyrosequencing followed by LNA-sequencing to further analyze this case. The IDH1 R132H mutation was not identified by any of these additional techniques. Consequently, based on the consistency of results obtained with PCR, Sanger sequencing, pyrosequencing and LNA-sequencing, it was likely that the mutation detected by IHC was a false-positive result.

**Table 5:** **Analysis of the IHC/PCR and PCR/Sanger sequencing discordant cases**

Case	IHC	IDH1/2 PCR	Sanger sequencing	Pyrosequencing	LNA-sequencing	Result	Conclusion
**# 1 (commercial)**	Pos	WT	WT	WT	WT	WT	IHC False-Pos
**# 2**	Pos	R132H	WT	-	-	R132H	Low Mutant Allele% (15%)
**# 3**	Pos	R132H	WT	-	-	R132H	Low Mutant Allele% (10%)
**# 4 (commercial)**	Neg	R132C	WT	R132C	-	R132C	Low Mutant Allele% (14%)
**# 5 (commercial)**	Neg	R132	WT	WT	WT	WT	PCR False-Pos*
**# 6**	Neg	R172	WT	WT	WT	WT	PCR False-Pos*

IHC: Immunohistochemistry; PCR: Polymerase chain reaction; WT: Wild-type; Pos: Positive; Neg: Negative; LOD: limit of detection.

*PCR test result close to LOD value.

**Figure 6:**
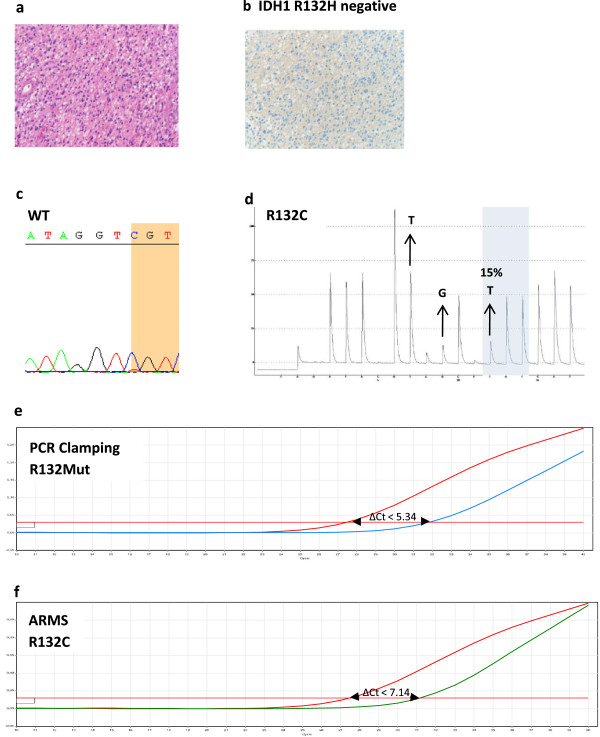
**Discordant case #4.** Case #4 was IDH1 R132H negative by IHC and was identified as wild-type by Sanger sequencing for *IDH1* codon 132. The IDH1/2 PCR assay identified an *IDH1* R132C mutation which was confirmed by pyrosequencing. **a** H&E slide. **b** IDH1 R132H IHC positive staining. **c** Sanger sequencing. **d** the pyrogram indicates the presence of the *IDH1* R132C mutation (TGT) at a low allele frequency (around 15%) suggesting that the sample contained low tumor cell content. **e** PCR-clamping: the difference between the IDH1 PCR-clamping mutation assay Ct (blue curve) and total assay Ct (red curve) is inferior to the cutoff value (ΔCt = 31.87 - 27.49 = 4.38 < 5.34) indicating the presence of a mutation within *IDH1* codon 132. **f** ARMS: the difference between the total assay Ct (red curve) and the *IDH1* R132C ARMS assay Ct (green curve) is inferior to the cutoff value (ΔCt = 31.20 – 27.49 = 3.71 < 7.14) indicating presence of the IDH1 R132C mutation.

Cases #2 and #3 were identified as IDH1 R132H positive according to both IHC and PCR testing, and negative with Sanger sequencing. Review of the IHC R132H FFPE slide by a second pathologist confirmed the unambiguous IDH1 R132H positive IHC result. Quantitative PCR analysis revealed a low mutant allele percentage of 15% and 10% in cases #2 and #3, respectively. These percentages were below the Sanger sequencing sensitivity threshold (typically around 20% [27, 28]). Consequently this technical limit was likely to prevent mutation detection in these particular cases by Sanger sequencing.Similarly, in case #4 identified as IDH1 R132C by PCR but WT by Sanger sequencing, a low mutant allele percentage (14%) was the probable source of conflicting results between these 2 tests as the mutation was also detected by pyrosequencing (Figure 6).

Cases #5 and #6 were found to be IDH1 R132 and IDH2 R172 PCR-positive respectively, but were not detected by IHC or Sanger sequencing. Pyrosequencing and LNA sequencing confirmed the absence of mutation in both cases, thereby indicating probable false PCR-positive results. The PCR Ct values of these 2 cases were close to the cut-off values of the respective mutations (data not shown). In order to eliminate a technical issue, PCR runs were repeated twice on cases #5 and #6 and the results indicated an IDH WT status. The first PCR results could therefore be considered as false-positive.

### Validation of rare IDH1/2 mutations using synthetic samples

As the likelihood of having a significant representation of rare *IDH1/2* mutations in the clinical cohort was low, synthetic samples representative of all 11 rare mutations were prepared. Each rare mutation was tested at 2 mutant allele percentages (30 and 40% of mutant DNA mixed with IDH WT genomic DNA). All mutations were correctly detected by the PCR assay in the 22 samples tested.

## Discussion

We developed a novel PCR assay for the detection of 12 *IDH1/2* mutations in one single step. A fast and reliable *IDH* mutation assay easy to implement in daily practice is highly desirable, given the diagnostic and prognostic significance of *IDH* mutations in diffuse glioma of low and intermediate grade (WHO grade II & III) as well as in secondary GBM (WHO grade IV). Testing for the presence of an *IDH1/2* mutation is now considered by international guidelines for glioma management [29, 30]. Very recently, the predictive use of *IDH* mutations has been confirmed in grade III glioma [14]. There is also an increasing interest in the development of IDH-targeted therapies. Newly developed mutated-IDH selective inhibitors have already demonstrated therapeutic effect in preclinical models [17] and an alternative approach whereby pathologic DNA-methylation is targeted, has led to tumor control in *IDH* mutant cells [18, 19]. Consolidation of these promising results in future clinical studies will imply identification of patients carrying specific *IDH* mutations with a reliable companion diagnostics test.

Currently, IHC covers the most prevalent *IDH1* mutation, and various molecular techniques are used to assess the IHC-negative cases, leading to a two-step diagnostic algorithm with possibly long timelines to complete a full *IDH* profiling. With the Sanger sequencing, multiple DNA-based assays have been adapted for the detection of *IDH* mutations, including restriction length fragment polymorphism gel electrophoresis, PCR and endonuclease restriction, allele-specific polymerase chain reaction, pyrosequencing, high resolution melting (HRM) or cold-PCR HRM [31–36].

These methods present pros and cons in terms of ease of use, rapidity, and sensitivity performance. The majority of published assays targets the most frequent *IDH1* R132 mutations but they are not designed to detect other rare ones including those within that same codon 132, the *IDH1* R100 mutations or the *IDH2* mutations. Therefore we developed an alternative assay for rapid (only 5 hours from DNA extraction to data acquisition) and comprehensive analysis of the 12 clinically *IDH1/2* mutations reported to date, with a single-step design compatible with easy implementation in routine clinical practice. Demonstration of high analytical and clinical performance of a new test is critical before implementation in a pathology laboratory. In this study, we validated the performance of this new IDH1/2 PCR assay on a large cohort of FFPE glioma samples collected less than 10 years ago and on synthetic samples mimicking all 11 rare *IDH1/2* mutations. Out of the 147 FFPE tested samples, the overall technical success rate of the PCR assay was 91%, reaching 100% on the 103 samples collected from academic centers. The technical success rate obtained on the remaining 43 commercial samples (DNA extraction failed from one sample) was lower (70%); this was probably due to a poor formalin fixation of this specific FFPE sample series, therefore impacting on the sample quality.

The assay showed a higher sensitivity than Sanger sequencing which typically detects levels as low as around 20% of mutant allele load. Without any mutant DNA enrichment before the IDH1/2-specific PCR, the test showed high analytical sensitivity with detection of mutant allele frequency, ranging from 0.6% for *IDH2* R172K to below 5% for all mutations except for the rare *IDH2* R172S one. Moreover, the precision study demonstrated that results for the mutation detection were highly reproducible and reliable including for mutant allele frequencies of 5%.

Out of the 133 clinical samples with a successful PCR IDH determination, *IDH1/2* mutations were detected in 54% of cases. The frequency of mutations by histological subtype was similar to previous reports from large series [5] with 13% of mutations identified in primary GBMs as opposed to 83% in the other subtypes. We further examined the PCR test accuracy by comparing, in a blind manner, the results obtained from the 133 clinical FFPE samples to the ones from IDH1 R132H-IHC and Sanger sequencing, which are the 2 main clinical diagnostic technologies recommended in the current algorithms for IDH testing. Overall concordance for *IDH1* R132H detection was 99% between PCR and IHC and 96% between PCR and Sanger sequencing. Positive concordances were high, with 98% between PCR and IHC and 100% between PCR and Sanger sequencing. The high concordance of the new PCR assay with the standard technologies demonstrated that the test could be used reliably for clinical diagnostics.

Regarding the agreement with IDH1 R132H-IHC, only one case was detected as negative discordant and this case was likely to be a false-positive IHC case as its analysis with 2 additional sensitive techniques confirmed its PCR and Sanger sequencing-identified IDH WT status. This highlights the need to ensure high quality in IHC testing when used for routine clinical diagnostic [37]. The number of cases (5 identified in total) with conflicting results from the IDH1/2 PCR assay and Sanger sequencing was less than 4%. Three of them could be explained by a low mutant allele content (<20%) which is below the Sanger sequencing sensitivity and therefore the mutation could not be detected using this technique. This further illustrates that if sensitivity of sequencing is generally not an issue when analyzing tumor samples with high content of neoplastic cells, it can become a limiting factor in the absence of macro-dissection for samples with a low tumor cellularity, leading to false negative results.

Overall, only 2 false-positive PCR results out of the 133 samples tested here were identified by comparison of IHC, Sanger sequencing, pyrosequencing and LNA-sequencing, which consistently established a WT IDH status for these 2 samples. Repeated PCR tests on these samples indicated a WT IDH status, supporting the hypothesis that the false-positive result from the first run might have come from an experimental error.

Additional synthetic samples were further analyzed in order to confirm the correct detection of all 11 rare mutations from the PCR assay and this analysis indicated 100% correct calls, further validating the performance of the assay.

Interestingly, a new dinucleotide deletion/insertion mutation has been reported since the design of this assay [38]. Since this newly identified mutation is localized within the codon 132 of the *IDH1* gene, the assay design and more specifically the clamping reaction, may allow its detection. It will be of interest to confirm this on synthetic samples reproducing this mutation and then on clinical FFPE samples if available.

## Conclusions

In summary, we have developed a new PCR-based assay which enables rapid and simple detection of the major *IDH1* R132H mutation and of 11 rarer *IDH1/2* ones in FFPE glioma samples. The test is designed as a single-step PCR procedure combining clamping and ARMS. We have established here the high sensitivity and reliability of this new assay and validated it on 171 FFPE clinical samples, indicating that the test fulfilled both analytical and clinical performance required for implementation in clinical practice. The PCR test could be used as a rapid and robust novel tool in the clinical assessment of diffuse gliomas as well as in selecting patients suitable for clinical trials.

## Electronic supplementary material

Additional file 1: Table S1: Sensitivity of the IDH1/2 PCR assay for the 12 detected and 3 identified *IDH1/2* mutations. **Table S2.** Precision results. **Table S3.** Analysis of Agreement between the IDH1/2 PCR assay and IDH1 R132H-IHC and Sanger sequencing. (PDF 350 KB)

Additional file 2: Figure S1: Sensitivity of the PCR 1DH1/2 assay for each mutation. Box plots depict the ΔCt values obtained at five mutant allele percentages (2, 5, 10, 15 and 20%) in a WT DNA background for the 12 mutations detected by PCR-clamping (A, C, E) and the 3 mutations identified by ARMS (B, D). Data were obtained from repeated and independent measurements as described in materials and methods. The red lines denote the determined LOD. (PDF 17 KB)
