# Supplementary material for: A new sensitive PCR assay for one-step detection of 12 IDH1/2 mutations in glioma
Source: Acta Neuropathol Commun. 2014 Jun 2;2:58. doi: 10.1186/2051-5960-2-58 (PMC4229941; doi:10.1186/2051-5960-2-58)
Supplement: Supplementary file 1 — Additional file 1: Table S1: Sensitivity of the IDH1/2 PCR assay for the 12 detected and 3 identified IDH1/2 mutations. Table S2. Precision results. Table S3. Analysis of Agreement between the IDH1/2 PCR assay and IDH1 R132H-IHC and Sanger sequencing. (PDF 350 KB) [file 40478_2014_132_MOESM1_ESM.pdf]

Table S1. Sensitivity of the IDH1/2 PCR assay for the 12 detected and 3 identified IDH1/2 mutations

Assay	Mutation	LOD	Assay cutoff *	Sensitivity (%)
R132HMut	R132H	6.87	6.87	0.78
R132CMut	R132C	7.14	7.14	1.19
R172KMut	R172K	8.49	8.49	0.61
R132Mut	R132H	5.50	5.34	2.32
	R132C	5.34		4.35
	R132L	5.42		2.30
	R132G	5.61		2.23
	R132S	5.42		2.75
	R132V	5.56		2.24
R172Mut	R172K	6.42	6.42	1.06
	R172G	6.58		3.00
	R172M	6.66		3.31
	R172S	6.42		14.93
	R172W	6.68		2.36
R100Mut	R100Q	4.65	4.65	3.45

LOD: Limit of Detection.

*A mutation is detected if ΔCt is less or equal to the LOD

Table S 2. Precision results

Assay	Sample	Mean ΔC_T	SD_R*	SD_{Run} †	SD_{Total} ‡	CV_{Total} (%)‡	Correct calls rate % (Frequency)
**R132C MUT**	WT	11.58	1.08	0.00	1.11	10	100% (78/78)
	5%	5.19	0.26	0.23	0.46	9	100% (76/76)
	10%	4.37	0.27	0.14	0.48	11	100% (78/78)
	30%	2.62	0.20	0.21	0.46	18	100% (78/78)
**R132H MUT**	WT	10.87	1.48	0.00	1.48	14	100% (78/78)
	5%	4.46	0.27	0.05	0.31	7	100% (78/78)
	10%	3.57	0.28	0.14	0.31	9	100% (76/76)
	30%	1.86	0.21	0.20	0.30	16	100% (72/72)
**R172K MUT**	WT	12.20	0.31	0.17	0.39	3	100% (66/66)
	5%	6.19	0.50	0.00	0.63	10	100% (76/76)
	10%	5.23	0.32	0.20	0.48	9	100% (76/76)
	30%	3.68	0.18	0.11	0.36	10	100% (76/76)
**R100 MUT**	WT	7.21	0.41	0.27	0.52	7	100% (70/70)
	5%	3.68	0.27	0.16	0.33	9	100% (76/76)
	10%	2.93	0.24	0.15	0.32	11	100% (76/76)
	30%	1.56	0.25	0.07	0.26	17	100% (76/76)
**R132 MUT**	WT	8.01	0.76	0.00	0.78	10	100% (152/152)
	R132H 5%	4.29	0.30	0.15	0.48	11	99% (151/152)
	R132C 5%	4.44	0.30	0.00	0.56	13	
**R132 MUT**	R132H 10%	3.49	0.27	0.22	0.46	13	99% (151/152)
	R132C 10%	3.69	0.27	0.23	0.53	14	
	R132H 30%	1.87	0.21	0.02	0.33	18	100% (152/152)
	R132C 30%	2.00	0.26	0.28	0.59	29	
**R172 MUT**	WT	9.47	0.91	0.87	1.45	15	100% (66/66)
	5%	4.45	0.35	0.12	0.56	13	100% (76/76)
	10%	3.55	0.29	0.02	0.53	15	100% (76/76)
	30%	2.05	0.18	0.15	0.47	23	100% (76/76)

WT: Wild-type; SD: Standard deviation; CV: Coefficient of variation.

* R: Repeatability; † Run: Between-run reproducibility; ‡ Total: Total precision (including inter-instrument, inter-operator, and inter-lot).

Table S 3. Analysis of Agreement between the IDH1/2 PCR assay and IDH1 R132H-IHC and Sanger sequencing

	Measure of agreement	% (Frequency)	95% CI
**IDH1/2 PCR *vs* IHC**	Overall percent agreement	99.0% (102/103)	[95.9 - 99.9]
	Positive percent agreement	98.4% (60/61)	[91.3 - 99.7]
	Negative percent agreement	100% (72/72)	[94.9 - 100]
**IDH1/2 PCR *vs* Sanger Sequencing**	Overall percent agreement	96.2% (128/133)	[91.5 - 98.4]
	Positive percent agreement	100% (67/67)	[94.6 - 100]
	Negative percent agreement	92.4% (61/66)	[83.5 - 96.7]

IHC: Immunohistochemistry; PCR: Polymerase chain reaction; CI: Confidence interval.
