# Supplementary figures and images for: A new sensitive PCR assay for one-step detection of 12 IDH1/2 mutations in glioma
Source: Acta Neuropathol Commun. 2014 Jun 2;2:58. doi: 10.1186/2051-5960-2-58 (PMC4229941; doi:10.1186/2051-5960-2-58)

(A) R132 PCR-clamping

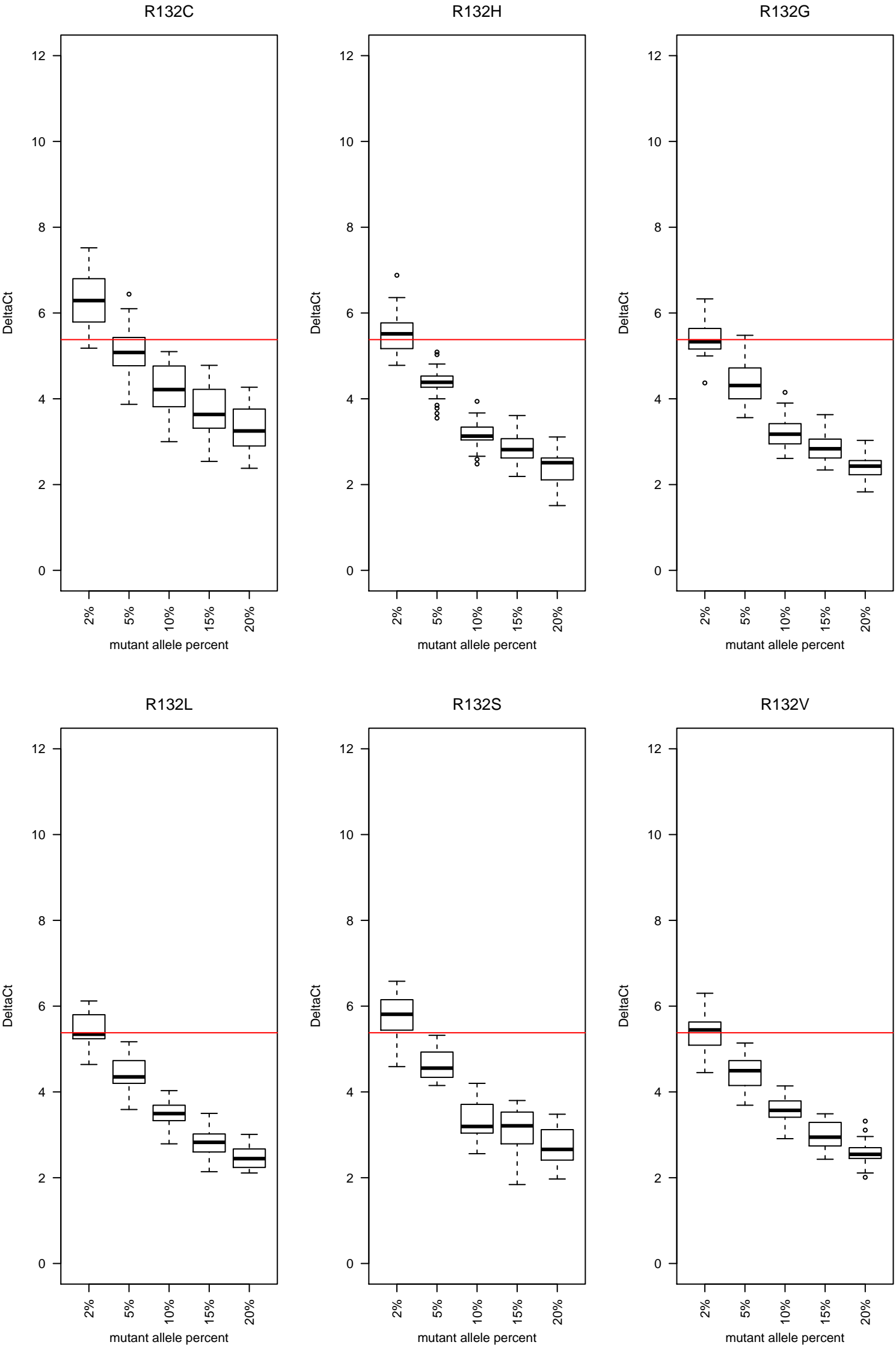

(B) R132 ARMS

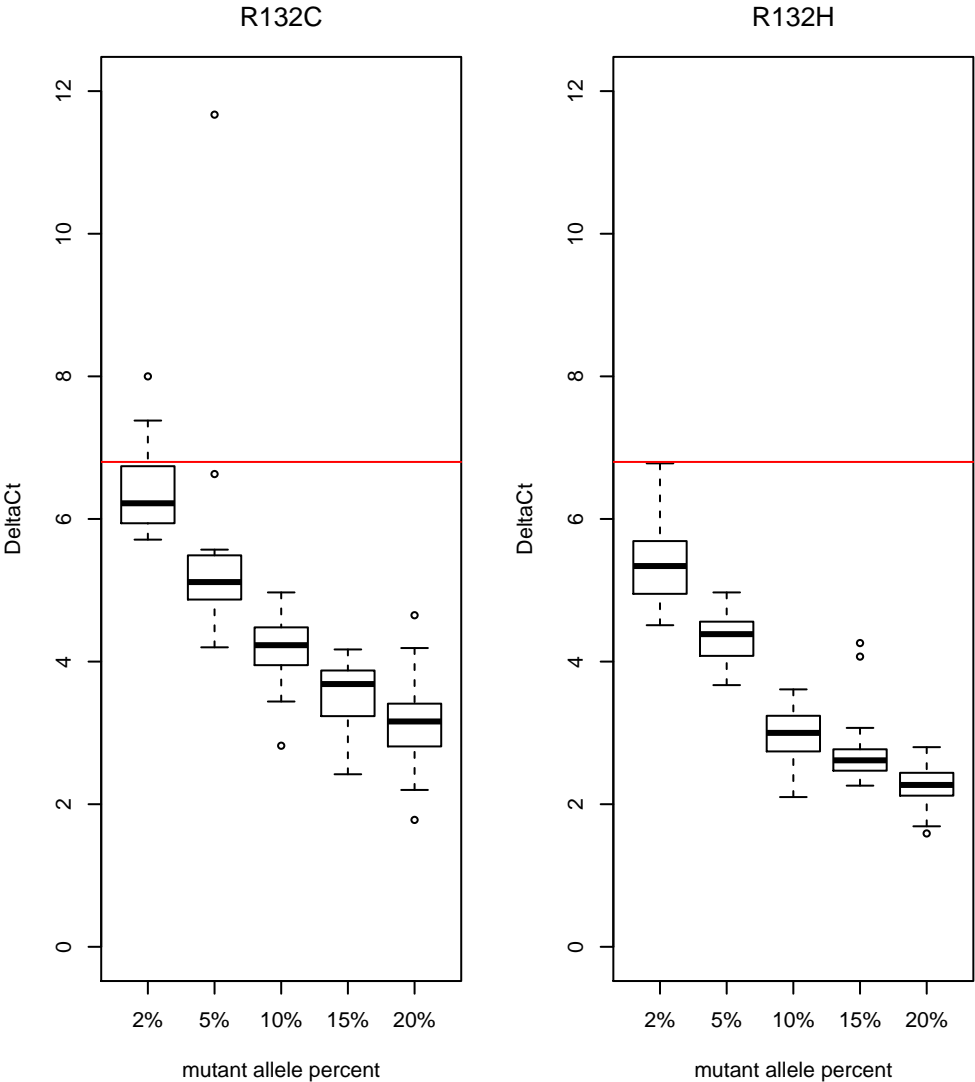

(C) R172 PCR-clamping

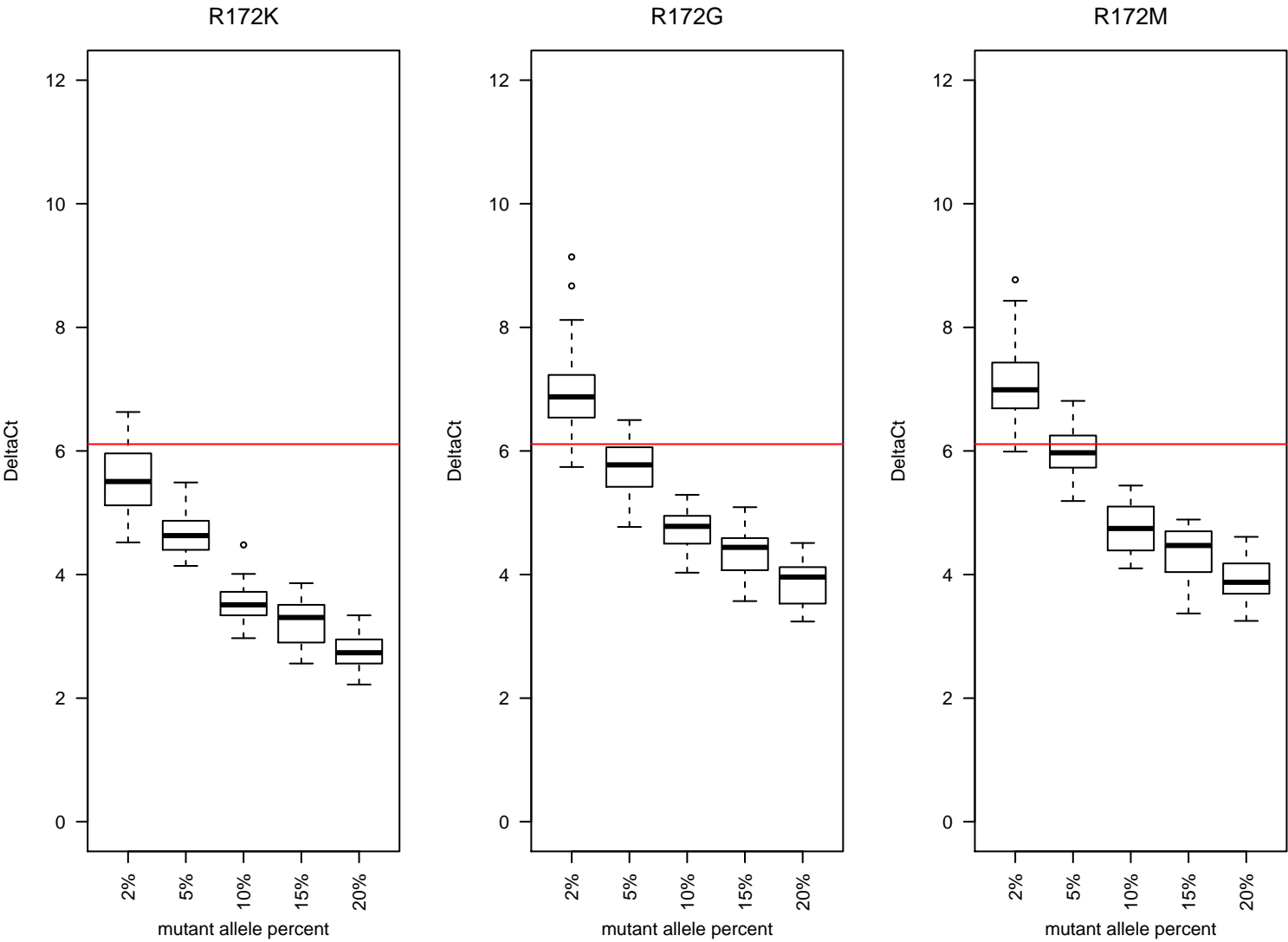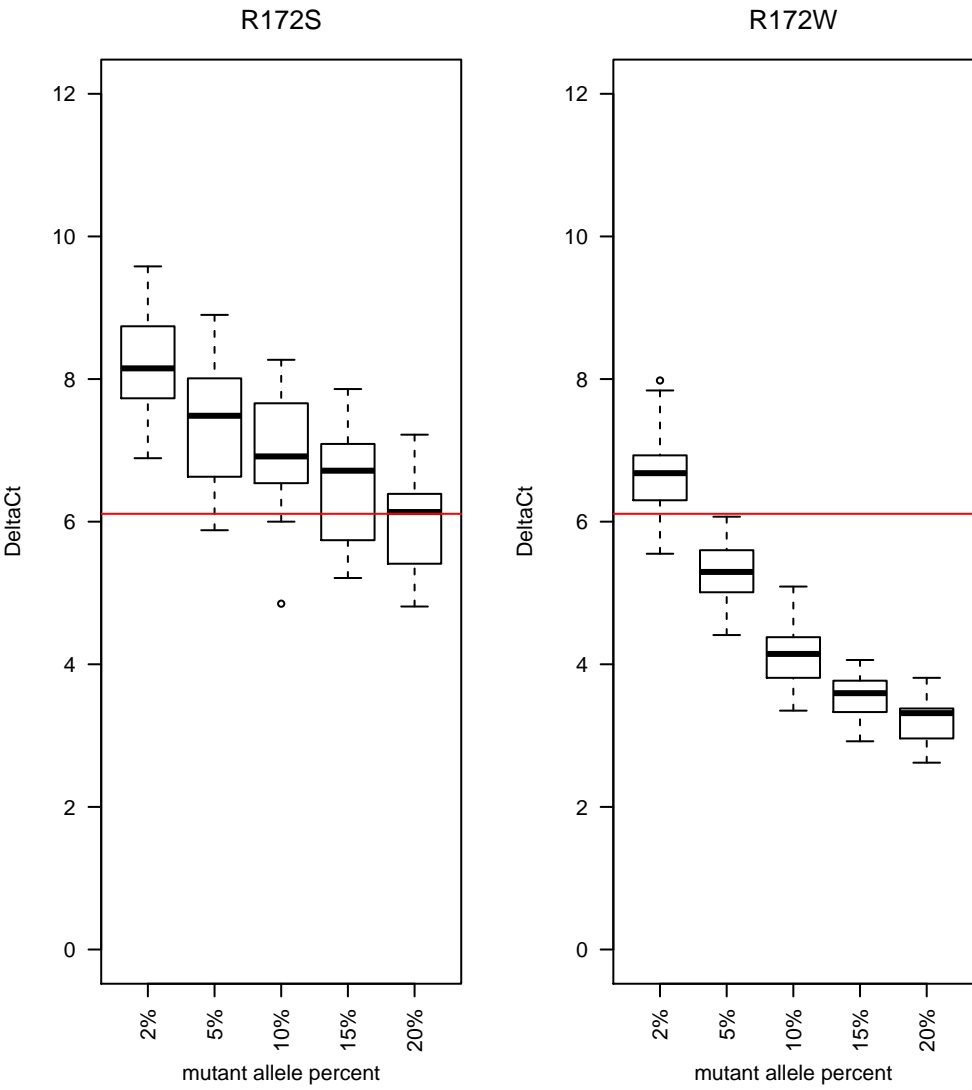

(D) R172 ARMS

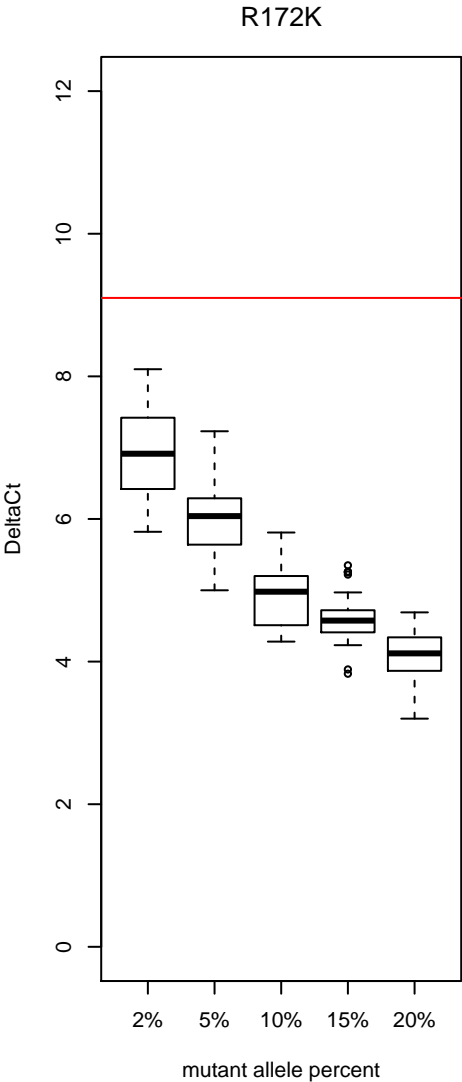

(E) R100 PCR-clamping

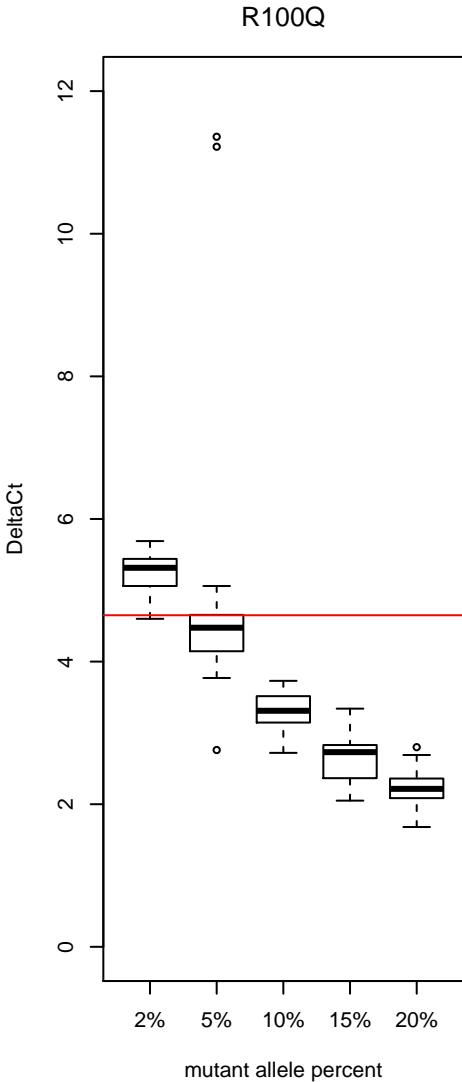

Supplement: Supplementary file 2 — Additional file 2: Figure S1: Sensitivity of the PCR 1DH1/2 assay for each mutation. Box plots depict the ΔCt values obtained at five mutant allele percentages (2, 5, 10, 15 and 20%) in a WT DNA background for the 12 mutations detected by PCR-clamping (A, C, E) and the 3 mutations identified by ARMS (B, D). Data were obtained from repeated and independent measurements as described in materials and methods. The red lines denote the determined LOD. (PDF 17 KB) [file 40478_2014_132_MOESM2_ESM.pdf]
